# Omics profiles used to evaluate the gene expression of *Exiguobacterium antarcticum* B7 during cold adaptation

**DOI:** 10.1186/1471-2164-15-986

**Published:** 2014-11-18

**Authors:** Hivana PMB Dall’Agnol, Rafael A Baraúna, Pablo HCG de Sá, Rommel TJ Ramos, Felipe Nóbrega, Catarina IP Nunes, Diego A das Graças, Adriana R Carneiro, Daniel M Santos, Adriano MC Pimenta, Marta SP Carepo, Vasco Azevedo, Vivian H Pellizari, Maria PC Schneider, Artur Silva

**Affiliations:** Laboratório de Polimorfismo de DNA, Instituto de Ciências Biológicas, Universidade Federal do Pará, Belém, Brasil; Laboratório de Ecologia de Micro-organismos, Instituto Oceanográfico, Universidade de São Paulo, São Paulo, Brasil; REQUIMTE-CQFB – Departamento de Química, Faculdade de Ciências e Tecnologia, Universidade Nova de Lisboa, Campus de Caparica, 2829-516 Caparica, Portugal; Laboratório de Venenos e Toxinas, Instituto de Ciências Biológicas, Universidade Federal de Minas Gerais, Belo Horizonte, Brasil; Laboratório de Genética Celular e Molecular, Instituto de Ciências Biológicas, Universidade Federal de Minas Gerais, Belo Horizonte, Brasil; Institute of Biological Sciences, DNA Polymorphism Laboratory, Federal University of Pará, 01 Augusto Corrêa st, 66075110 Belém, PA Brazil

**Keywords:** *Exiguobacterium antarcticum*, Psychrotrophic, Proteomic, RNA-Seq, Gene expression

## Abstract

**Background:**

*Exiguobacterium antarcticum* strain B7 is a Gram-positive psychrotrophic bacterial species isolated in Antarctica. Although this bacteria has been poorly studied, its genome has already been sequenced. Therefore, it is an appropriate model for the study of thermal adaptation. In the present study, we analyzed the transcriptomes and proteomes of *E. antarcticum* B7 grown at 0°C and 37°C by SOLiD RNA-Seq, Ion Torrent RNA-Seq and two-dimensional difference gel electrophoresis tandem mass spectrometry (2D-DIGE-MS/MS).

**Results:**

We found expression of 2,058 transcripts in all replicates from both platforms and differential expression of 564 genes (absolute log2FC ≥1, P-value <0.001) comparing the two temperatures by RNA-Seq. A total of 73 spots were differentially expressed between the two temperatures on 2D-DIGE, 25 of which were identified by MS/MS. Some proteins exhibited patterns of dispersion in the gel that are characteristic of post-translational modifications.

**Conclusions:**

Our findings suggest that the two sequencing platforms yielded similar results and that different omic approaches may be used to improve the understanding of gene expression. To adapt to low temperatures, *E. antarcticum* B7 expresses four of the six cold-shock proteins present in its genome. The cold-shock proteins were the most abundant in the bacterial proteome at 0°C. Some of the differentially expressed genes are required to preserve transcription and translation, while others encode proteins that contribute to the maintenance of the intracellular environment and appropriate protein folding. The results denote the complexity intrinsic to the adaptation of psychrotrophic organisms to cold environments and are based on two omic approaches. They also unveil the lifestyle of a bacterial species isolated in Antarctica.

**Electronic supplementary material:**

The online version of this article (doi:10.1186/1471-2164-15-986) contains supplementary material, which is available to authorized users.

## Background

Prokaryotes are able to adapt to a wide range of environmental conditions, including extreme variations in temperature, pressure, salinity, pH and radiation [1, 2]. As temperature interferes with the cell structure and function, it is considered one of the most life-limiting physical parameters and thus acts as a determinant of the distribution of living organisms across the earth [2, 3].

Most of the Earth’s biosphere (more than 80%) is cold. Large land areas are permanently frozen or unfreeze for only a few weeks in the summer, while the temperature of 90% of the ocean water is lower than 5°C. Representatives of all three life domains (Bacteria, Archaea and Eukarya) have successfully colonized these cold ecosystems [4]. Microorganisms adapted to cold conditions are classified as psychrophiles when their optimal temperature for growth is approximately 15°C or lower and as psychrotrophic when their optimal temperature for growth is above that level [2, 5]. Free-living bacteria constantly monitor the environmental temperature and can detect temperature changes by thermosensors, which are biomolecules sensitive to such changes [6, 7]. At low temperatures, several adaptive mechanisms are activated to maintain membrane fluidity, transport, transcription, translation, cell division, metabolism and enzyme activity and to avoid intracellular ice formation [8, 9].

The compatibility of organisms with their habitat temperature is determined by their genetic architecture [3] and their ability to respond to environmental variations by changes in gene expression. However, the sets of genes required for adaptation to low temperatures have not yet been fully identified [10]. To address this gap, several genomes, transcriptomes and/or proteomes of cold-adapted microorganisms have been investigated [11, 12].

Omic research has been recently advanced due to the introduction of next-generation sequencing (NGS) technologies and increasingly sensitive proteomic techniques, such as two-dimensional difference gel electrophoresis tandem mass spectrometry (2D-DIGE-MS/MS), which provide a wide variety of data [13]. The RNA-Seq method, in which complementary DNA fragments are sequenced using NGS [14], opened new horizons for the analysis of the prokaryotic transcriptome as it allowed the quantification of differential expression as well as an understanding of the bacterial RNA type diversity and its regulatory mechanisms. The transcriptomes of several pathogenic or bacteria with environmental and/or biotechnological relevance were sequenced by RNA-Seq under various conditions, and the results were published [15–17]. However, the microbial adaptation to extreme environmental conditions was poorly investigated by this method.

*Exiguobacterium antarcticum* strain B7 was isolated from a biofilm in Ginger Lake, King George Island, Antarctica (62°10’S and 058°25’W), and its genome was sequenced [18]. This species is a relevant model for the study of the microbial ability to survive and proliferate within a wide temperature range, as it grows in temperatures ranging from -3°C to 42°C [19], with an optimal growth temperature of 37°C [20].

In the present study, we identified the main genes involved in *E. antarcticum* B7 adaptation to cold and defined the global gene expression in response to temperatures of 37°C and 0°C through the generation of omic data. We confirmed the relevance of some genes previously reported in the literature to thermal adaptation and also describe new findings that allow a better understanding of the lifestyle of this psychrotrophic organism.

## Methods

### Bacteria and growth conditions

Bacterial cultures were diluted to optical density 0.04 at 600 nm (OD600) and grown in Tryptone Soy Broth (TSB, HiMedia, India) at 0°C or 37°C under constant agitation at 210 rpm until reaching OD600 0.4–0.5, which corresponds to the middle of the exponential growth phase. At this point, total RNA and protein extractions were performed separately. For RNA extraction, a culture volume (100 ml) was immediately mixed with an equal volume of RNAlater® (Ambion, USA). For protein extraction a 500 ml culture volume was used. Biological triplicates were prepared for each omic experiment.

### RNA extraction, enrichment of mRNA and RNA-Seq

Aliquots of 2 ml were centrifuged for sediment the cells, at 5000 g and 4°C for 10 minutes. The bacterial total RNA was extracted using *ChargeSwitch® Total RNA Cell Kits* (Invitrogen, USA) according to the manufacturer’s instructions, and adding a mechanical lysis step using a *Precellys*^*©*^ grinder (Bertin Technologies, France). The total RNA amount was measured by fluorometry using a *Qubit™ - Quant-iT™ RNA Assay Kit* (Invitrogen, USA). Messenger RNA was enriched using *Ribominus™ Transcriptome Isolation Kit* (*Yeast and Bacteria*) (Invitrogen, USA), and the amount of recovered RNA was measured as described above.

The enriched RNA samples were used to build strand-specific RNA-Seq libraries. Two libraries, one at each experimental condition (0°C and 37°C, designated R1-0 and R1-37, respectively), were prepared using a SOLiD™ Total RNA-Seq Kit. Fifty-base pair (bp) fragments were sequenced using the SOLiD™ 3 Plus system according to the manufacturer’s instructions (Applied Biosystems, USA). Two replicates from each experimental condition (0°C and 37°C, designated as R2-0 and R3-0 and as R2-37 and R3-37, respectively) were employed in the preparation of fragment libraries using Ion Total RNA-Seq Kit and sequenced using 316 chip in an Ion Torrent Personal Genome Machine™ (Life Technologies, USA). All the procedures were performed according to the manufacturer’s instructions.

### Transcriptomic analysis and validation

The quality reads were mapped against the *E. antarcticum* B7 annotated genome [Genbank: CP003063] using CLC Genomics Workbench™ (CLC Bio^©^, Denmark). The reads obtained using SOLiD™ (R1-0 and R1-37) were cut to 35 bp using an in-house developed script and filtered (mean quality value – QV <15) using the software Quality Assessment [21]. In the case of the reads obtained using the Ion Torrent platform (R2-0, R3-0, R2-37 and R3-37), the software CLC Genomics was configured to discard reads shorter than 35 bp or longer than 150 bp.

The reads that did not map to noncoding RNA (ncRNA), rRNA or tRNA regions, were counted by CLC Genomics software and included in the statistics of possible coding regions as "reads per kilobase million" (RPKM) [22]. For the SOLiD datasets, two measurements of expression were performed and compared; in one measurement, the multireads were discarded, while in the other, they were counted according to the criteria depicted in Additional file 1: Figure S1.

To assess the differential expression between the two investigated temperatures, the RPKM obtained in the previous step were analyzed using R package DEGseq [23] as follows: The RPKM values of the SOLiD datasets (R1-0 vs. R1-37) and the mean RPKM values of each experimental condition corresponding to the datasets sequenced using Ion Torrent (R2,3-0 vs. R2,3-37) were compared, thus generating log base 2-fold change values (log2FC) and P-values.

### Protein extraction and two-dimensional analysis

The 2D-DIGE technique was used to characterize the protein expression profile. For protein extraction, cell lysis was performed using FRENCH Press (Thermo Scientific, USA) after the cells were washed using 10 mM Tris–HCl buffer at pH 7.6 supplemented with 1 mM DTT. The soluble proteins were then obtained by centrifugation at 8,000 g for 30 minutes, which was followed by ultracentrifugation at 100,000 g for 90 minutes. Wide-range pH strips were first used to identify the pH values associated with the greatest protein concentration (Additional file 2). To perform 2D-DIGE, the samples were quantified and 120 μg of protein extract was precipitated with methanol/chloroform and resuspended until a final concentration of 3 μg μl^-1^ was achieved. In each sample, 54 μg of protein was labeled with 400 pmol Cy3 or Cy5, and an internal standard containing all the samples from all replicates was labeled with Cy2. Each replicate was mixed and applied to the same gel following dilution with rehydration buffer (7 M urea, 2 M thiourea, 2% CHAPS and 0.5% immobilized pH gradient (IPG) buffer) and 50 mM DTT. Strips of 18 cm and pH 4–7 were used for isoelectric focusing in an Ettan™ IPGphor II (GE Healthcare, Sweden) following a five-step protocol: 100 V for one hour, 500 V for two hours, 1,000 V gradient for two hours, 10,000 V gradient for three hours and 10,000 V step until reaching 60000 V h^-1^ with a constant current of 75 μA per strip. Following the isoelectric focusing, second dimension separation was performed using an Ettan™ DALTsix electrophoresis unit (GE Healthcare, Sweden). The resulting gels were digitized using an Ettan™ DIGE Imager (GE Healthcare, Sweden), and the images were analyzed using Image Master 2D Platinum v.7.0 (GE Healthcare, Sweden). The protein spots exhibiting ±2-fold differences in relative volume were considered differential spots. Only the differential spots that exhibited significance in ANOVA (p <0.05) were taken into consideration.

Following the analysis of expression, the differential spots were picked from preparatory gels containing 450 μg of protein each using Ettan™ Spot Picker (GE Healthcare, Sweden). The proteins were digested with trypsin, 20 ng μl^-1^ (Promega, USA), at 58°C for 30 minutes. The peptides were then extracted from the gel and placed on an AnchorChip 600 plate for use in a MALDI-TOF/TOF AutoFlex III™ mass spectrometer (Bruker Daltonics, USA), in positive reflector mode, for identification. The resulting MS and MS/MS (LIFT mode) spectra were analyzed using Mascot ( http://www.matrixscience.com) and compared to genomic data corresponding to that of the group Firmicutes deposited at the National Center for Biotechnology Information (NCBI) nr database ( http://www.ncbi.nlm.nih.gov).

## Results and discussion

### Absolute expression analysis and the detection of differentially expressed genes by RNA-Seq

*E. antarcticum* B7 transcriptomes were sequenced after culture growth at 0°C and 37°C. One replicate from each experimental condition (R1-0 and R1-37) was sequenced using the SOLiD™ system (Applied Biosystems, USA), and two additional replicates from each experimental condition (R2-0, R3-0, R2-37 and R3-37) were sequenced using the PGM Ion Torrent platform (R2-0, R3-0, R2-37 and R3-37) to validate the thermo-specific expression profile of *E. antarcticum* B7 (Table  1 and Additional file 3: Table S1). In the step of library preparation, the ribosomal RNAs (rRNA 16S and 23S subunits) were depleted. Thus, the ncRNA reads were filtered from our data set, and the remaining reads that aligned to mRNA regions were used for the gene expression estimate (Table  1 and Additional file 3: Table S1).

**Table 1:** **RNA-Seq dataset statistics**

	Total reads dataset*	Quality dataset**	rRNA/tRNA	mRNA	Sequencing platform
**R1-0**	79,405,140	65,080,480	26,970,143	10,989,871	SOLID
**R1-37**	76,980,212	63,827,885	29,981,464	4,497,647	SOLID
**R2-0**	1,032,011	897,047	443,740	32,354	ION
**R3-0**	1,073,341	963,777	672,607	34,969	ION
**R2-37**	1,996,177	1,701,101	1,271,962	55,633	ION
**R3-37**	1,535,558	1,388,771	1,010,305	42,129	ION

*Number of reads generated in the sequencing of each replicate.

**Number of reads remaining after quality treatment that were used in mapping.

We analyzed the expression levels of 2,851 annotated regions of the *E. antarcticum* B7 genome, of which 2,773 were protein-coding genes and 78 were pseudogenes. Relative to the R1-0 and R1-37 datasets, the results obtained with and without the inclusion of multireads showed that the latter represented only 2% (221,912/10,989,871) and 0.36% (16,121/4,497,647) of the total number of aligned reads and that they were concentrated within a few of the assessed regions. For that reason, the final RPKM values of the vast majority of transcripts corresponding to either temperature (0°C or 37°C) are derived from reads that were mapped in one single region (0% of multireads). However, the presence of transcripts whose expression fully depended on the multireads (100% of multireads) is noteworthy (Additional file 1: Figure S2). Some of these transcripts corresponded to genes conserved in the *E. antarcticum* B7 genome and are relevant for thermal adaptation, such as genes encoding cold-shock proteins. Similar results for the multireads were found in the other replicates (data not shown). Thus, we considered the results that used the multireads in the RNA-Seq analysis. This type of approach is scarcely reported in the literature [22, 24]; however, as our results show, the multireads exerted minimal interference on the transcriptome RPKM calculation and were only relevant in the calculation of the RPKM of extremely conserved genes, such as those encoding cold-shock proteins. The relevance of the inclusion of the multireads became evident when we performed the differential proteome analysis. The greatest relative volume values in the DIGE gels corresponded to cold-shock proteins (which were 32 times more expressed at 0°C than at 37°C). This finding validates the use of multireads in the calculation of the RPKM of those genes.

**Figure 1:**
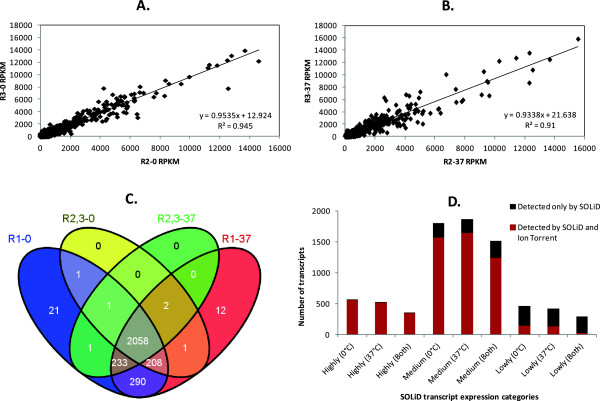
**Absolute expression analysis by SOLiD RNA-Seq and Ion Torrent RNA-Seq.** The upper panels describe the correlation between the biological replicates sequenced on the Ion Torrent platform at temperatures of 0°C **(A)** and 37°C **(B)**. **(C)** Venn diagram showing the total number of expressed regions in each replicate set. **(D)** Correlation between the expression levels of transcripts detected by SOLiD (Highly expressed, RPKM ≥500; Moderately expressed, 10 ≤ RPKM <500; Lowly expressed, 0 < RPKM <10) and those detected by Ion Torrent at each or both temperature conditions.

The biological replicates obtained using the Ion Torrent platform proved to be highly reproducible under both temperatures (Figure 1A and 1B) (R^2^ > 0.9). A comparison of the datasets from both platforms showed that no expressing region appeared in the results obtained using the Ion Torrent platform only, i.e., all regions were also detected in at least one of the libraries sequenced using SOLiD (Figure  1C). In addition, the efficiency of transcript detection by the Ion Torrent platform was proportional to that of the SOLiD platform (Figure  1D). The Venn diagram in Figure 1C provides an overall view of the total number of analyzed regions that were expressed (with one or more mapped reads, RPKM ≠ 0) in each set of replicates. SOLiD RNA-Seq (R1-0 and R1-37) covered 99% (2,828/2,851) of those regions, and most transcripts (98%, 2,789/2,828) were expressed at both temperatures. The percentage of regions covered by Ion Torrent RNA-Seq was 87.86% (2,505/2,851), and 82.27% (2,061/2,505) of them were expressed at both temperatures.

The expression levels of 23 regions were zero in all sequenced replicates. In total, 16 of these regions were annotated in databases as genes encoding hypothetical proteins with no predicted function, and five were annotated as pseudogenes. We also found transcripts that mapped in regions predicted to be pseudogenes. Although the occurrence of pseudogene transcription is reported in the literature, these regions may have been mistakenly assembled and/or erroneously annotated in bacterial genomes [24, 25].

Among the 2,020 genes expressed in all the replicates, those that met the following criteria were considered to be differentially expressed: (i) genes with the same log2FC value sign, i.e., those that appeared as up- (+) or downregulated (-) in the results of both platforms, and (ii) genes with a log2FC absolute value ≥1 and a P-value <0.001. A total of 564 genes met both criteria and, therefore, was considered to be differentially expressed by *E. antarcticum* B7 between the two temperatures.

### Protein expression profile on 2D-DIGE-MS/MS

**Figure 2:**
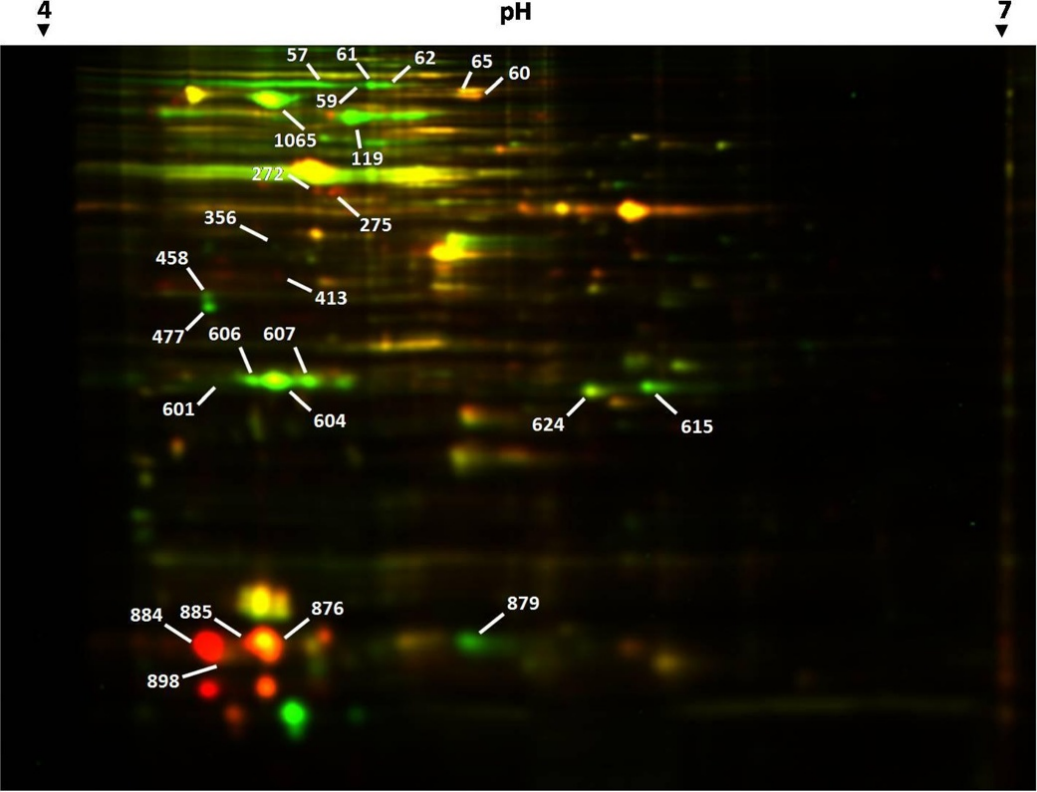
**Differential proteome gel of** ***E. antarcticum*** **B7 grown at 0°C and 37°C.** Spots in red correspond to bacteria grown at 0°C and labeled with Cy5, while those in green correspond to bacteria grown at 37°C and labeled with Cy3. The spots identified by MS/MS are indicated by blank arrows with the corresponding match ID.

Analysis by 2D-DIGE detected 73 spots that were differentially expressed between 0°C and 37°C (Figure  2), of which 48 were downregulated and 25 were upregulated at the colder temperature (Figure  2 and Additional file 3: Table S2). These differential spots were recovered from the preparative gels, and 25 of them were identified. These spots are indicated by their Match ID in Figure  2. These 25 spots corresponded to 13 different proteins. The relative differential expression range varied from -18.2498 (spot 952) to +33.4498 (spot 931). The expression levels of 10 of the 13 identified proteins were corroborated by the RNA-Seq results (Additional file 3: Table S2); however, the gene expression levels differed between the techniques (fold change vs. relative volume).

### Influence of temperature on *E. antarcticum*B7 gene regulation

**Figure 3:**
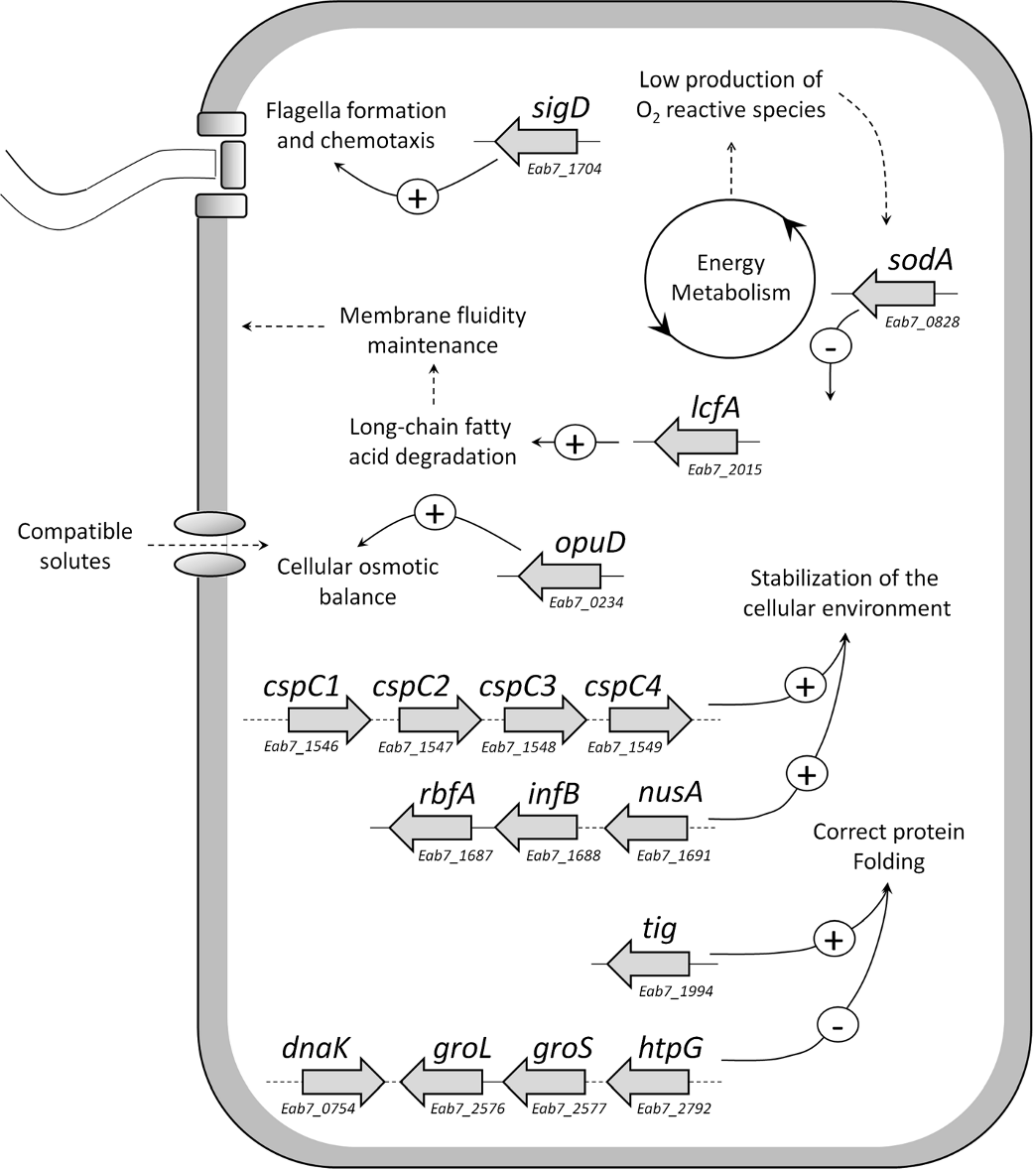
**Main differentially expressed genes and effects observed on the** ***E. antarcticum*** **cells grown at both temperature conditions.** The arrows exhibiting a plus sign indicate increased expression, and the arrows with a minus sign indicate reduced expression at 0°C. The locus tag of each gene is indicated below their representation. Genes clustered with full lines indicate operon arrangements, and those linked by dotted lines exhibit variable distances in the genome but perform similar cell functions.

The transcriptome results obtained by the two platforms relative to all *E. antarcticum* B7 genes and all the proteins identified by MS/MS are described in Additional file 3: Table S1 and Table S2. The main molecular mechanisms involved in the adaptation of *E. antarcticum* B7 described in the present study are summarized in Figure  3, and their relevance to culturing at cold conditions is more thoroughly addressed below.

#### Cold-shock proteins

Cold-shock proteins (Csp) homologues possess RNA chaperone activity thus are able to destabilize the secondary RNA structures formed as a result of exposure to cold that might impair transcription and translation [7]. Nine classes of Csp are described and classified according to their homologues found in the bacterial model *Escherichia coli. E. antarcticum* B7 has six genes that encode homologues of the main cold-shock proteins (Csp). All of them are conserved, are 201 bp long and have the nucleic acid-binding domain characteristic of this protein family [26]. Due to these characteristics, we had to take the *multireads* alignment into consideration to estimate these genes expression levels in a satisfactory manner (Additional file 4: Table S3). As a consequence, we detected these genes in all the replicates, and all were differentially expressed (Table  2). Four Csp genes were upregulated in the colder condition, while two other Csp genes were downregulated (Table  2). Different levels of expression of multiple Csp homologues of the same organism are reported in the literature [27]. *Psychrobacter arcticus* has two Csp homologues, one of which was highly expressed across the full temperature range, while the other was downregulated at -6°C [28]. In *Pseudomonas putida*, the transcriptomes analyzed by RNA-Seq have detected the expression of five Csp homologue proteins, three of which were upregulated and two of which were downregulated upon a reduction in temperature [29].

**Table 2:** **Differential expression of some significant** ***E. antarcticum*** **B7 genes between the temperatures 0°C and 37°C**

		SOLiD (R1-0 vs. R1-37)	Ion torrent (R2,3-0 vs. R2,3-37)
Gene ID	Product	log _2_FC	log _2_FC
**Cold shock response**			
Eab7_1546	Cold shock protein	2.61	1.94
Eab7_1547	Cold shock protein	3.6	2.16
Eab7_1548	Cold shock protein	2.65	2.30
Eab7_1549	Cold shock protein	2.59	2.46
Eab7_2272	Cold shock protein	-2.8	-1.06
Eab7_2747	Cold-shock DNA-binding domain protein	-1.84	-1.28
*cshA*	DEAD-box ATP-dependent RNA helicase CshA	2.74	2.38
*cshB*	DEAD-box ATP-dependent RNA helicase CshB	3.37	2.92
**Transcription, translation and regulation**			
*nusA*	Transcription elongation protein NusA	2.77	3
*nusG*	Transcription antitermination protein nusG	2.59	2.18
*infB*	Translation initiation factor IF-2	1.95	2.37
*infC*	Translation initiation factor IF-3	2.34	1.39
*rbfA*	Ribosome-binding factor A	1.98	2.46
*sigW*	RNA polymerase sigma factor SigW	-2.26	-1.6
*sigD*	RNA polymerase sigma-D factor	2.6	2.14
**Protein folding**			
Eab7_0447	Heat shock protein DnaJ domain protein	-3.31	-1.78
*clpB*	Chaperone protein ClpB	-3.27	-1.91
*grpE*	Protein grpE	-2	-1.6
*dnaK*	Chaperone protein DnaK	-2.24	-1.61
*Tig*	Trigger factor	1.49	0.93
*groL*	60 kDa chaperonin	-3.11	-2.12
*Gros*	10 kDa chaperonin	-3,21	-2
*htpG*	Chaperone protein htpG	-5.36	-3.62
**Cell membrane adaptations**			
*lcfA*	Long-chain-fatty-acid—CoA ligase	1.85	1.16
Eab7_0234	Choline/carnitine/betaine transporter	3.46	7
**Energy metabolism**			
Eab7_0571	Cytochrome c oxidase subunit II	-7.54	-9.6
*cbaB*	Cytochrome c oxidase subunit I	-6.02	-9.26
*ctaE*	Cytochrome c oxidase subunit 3	-1.72	-1.99
*ctaD*	Cytochrome c oxidase subunit 1	-1	-0.6
*ctaC*	Cytochrome c oxidase subunit 2	-1.68	-1.32
**Pyruvate metabolism – anaerobic**			
*pflA*	Pyruvate formate-lyase activating enzyme	-6.75	-6.7
*pflB*	Pyruvate formate-lyase	-7.4	-7.2
*Ldh*	L-lactate dehydrogenase	-3.12	-4
**Pyruvate metabolism – aerobic**			
*pdhD*	Dihydrolipoyl dehydrogenase	1.1	1.54
*pdhC*	Dihydrolipoyllysine-residue acetyltransferase component of pyruvate dehydrogenase complex	1.76	2

In *E. antarcticum* B7 proteome, one cold-shock protein, Csp1, was detected and identified by MS/MS in higher levels of expression at 0°C. It was found in four different gel spots, one of which exhibited a relative volume 32 times higher at 0°C than at 37°C (spot 884). That pattern of dispersion was most likely due to post-translational modifications. Modified proteins might be easily detected and identified using gel-based methods [30]. The displacement to the left of that same protein (spots 884 and 885) is characteristic of modifications in the pI value (Figure  2). That same behavior was found for the *Bacillus subtilis* cold-shock protein CspB and was attributed to the addition of a formyl group to the molecule [31]. This finding shows that Csp1 is the most abundant of the six cold-shock proteins, denoting its relevance for cell adaptation to cold.

Recently, a study demonstrated that in *E. coli* the CspC intracellular levels are inversely related to the concentration of the heat shock protein GroESL [32]. In the *E. antarcticum* B7 the genome sequence of the main cold shock protein described (Csp1) shows higher identity (85%) with the CspC class of *Bacillus subtilis*. In our data, the concentration of Csp1 also increases at 0°C followed by a decrease in the concentration level of the protein GroESL. This observation was corroborated by the two omics approaches used here. CspC protein acts by stabilizing the transcripts of several genes including the alternative sigma factor *rpoS* gene, which regulates several other genes for survival under stress conditions [33]. This is an expected response, since psychrotrophic organisms, such as *E. antarcticum* B7, are only adapted to low temperatures, requiring more complex molecular adaptation processes then those observed for psychrophilic bacteria. However, different Csp homologues also might be involved in different cell functions or types of stress, e.g., nutritional, osmotic and oxidative stress [34–36].

As a part of the cold shock response, DEAD-box RNA helicases might contribute to resolving secondary RNA structures, thus allowing for their efficient translation and later degradation [37]. In the present study, we found the overexpression of two genes encoding homologues of those proteins at 0°C compared to 37°C (Table  2). Similar results were reported in *Exiguobacterium sibiricum*, *P. putida*, *Bacillus cereus* and *P. arcticus*; in the latter two organisms, increased sensitivity to cold was found in deleterious mutants of those proteins [28, 29, 38, 39].

#### Transcription, translation and regulation

Among the most abundant mRNA species in *E. antarcticum* transcriptomes, those that encode *elongation factor* (Ef-Tu) and ribosomal proteins stand out; among the latter, several components of the 30S and 50S subunits were induced in the cold condition (Additional file 3: Table S1). Several genes encoding transcription and translation factors that might contribute to RNA and protein synthesis in the cold were overexpressed at 0°C compared to 37°C, including the *transcription factors* NusA and NusG, the *translation initiation factors* IF-2 and IF-3 and the *ribosome binding factor* RbfA (Table  2). Similar gene expression changes in response to cold were also found in other microorganisms, such as *E. sibiricum* [39], *P. arcticus* [28], *P. putida* [29] and *M. catarrhalis* [27].

We found differential expression of two genes encoding alternative sigma factors: *sigW* was repressed, and *sigD* was induced by cold (Table  2). An alternative sigma factor might be required by RNA polymerase holoenzyme to alter the expression of some genes or specific prokaryotic regulons simultaneously. In *Bacillus subtilis*, the regulon SigW contains genes associated with cell wall-related functions and adaptation to alkaline shock [40, 41], and the gene *sigW* was found to be expressed more lowly at cold [42]. Similarly, the gene *sigD,* which is related to a regulon of genes involved in flagellar synthesis, is one of 31 genes included in an operon, all of which participate in flagellar synthesis and bacterial chemotaxis [43]. All those genes comprising the SigW regulon, including the *sigW* gene, were upregulated at cold (Additional file 4: Table S4). These findings suggest that bacterial motility might be significant at low temperatures and that these genes, which were annotated as organized in an operon in the *E. antarcticum* B7 genome, are expressed as a polycistronic mRNA molecule.

Furthermore, flagellar synthesis in *Planococcus halocryophilus* is favorable at low temperatures (-15°C) [12]; however, in *E. sibiricum,* which is a closely related species, the genes involved in flagellar synthesis were repressed at -2.5°C compared to 28°C [39].

#### Protein folding

In the *E. antarcticum* response to cold, only Trigger factor-TF was overexpressed (Table  2). This protein has been recently described as the primary chaperone in *Pseudoalteromonas haloplanktis* grown at low temperatures and is involved in protein folding or refolding [44]. The remainder of the *E. antarcticum* B7 chaperones were underexpressed at 0°C in both the transcriptome and the proteome (Table  2 and Additional file 3: Table S2). These proteins were frequently reported to be repressed in the cold and to participate in the heat shock response [39, 44–46].

#### Cell membrane adaptations

Among the cell changes needed for microbial adaptation to cold, those involving the cell membranes are some of the most relevant and best documented. According to the available reports, an increase in the amount of short- and branched-chain unsaturated fatty acids seems to contribute to the maintenance of cell membrane fluidity and, consequently, function at low temperatures [11]. In *E. sibiricum* and *P. arcticus,* the expression of a gene associated with fatty acid desaturation was found to increase in the cold, corresponding to temperatures of -2.5°C vs. 28°C and -6°C vs. 17°C, respectively [28, 39]. In *E. antarcticum,* we did not find a consistent increase of the expression of the desaturase enzyme-encoding gene (*desA*) at 0°C. This enzyme is activated by the two-component system DesK-DesR [47]. DesK is a histidine kinase coupled to the membrane that senses the change in membrane fluidity and activates the regulatory protein DesR, which binds to the promoter region of the *desA* gene, inducing desaturation of fatty acids. Evaluating the genome of *E. antarcticum* B7, we observed that the *desK* gene is truncated, which would explain the lack of response of the desaturase *desA* to low temperature. However, the notorious ability to adapt to cold environments leads us to believe that other pathways are used for this purpose. For example, we found the overexpression of the gene *lcfA* (Table  2), the product of which is related to long-chain fatty acid degradation [48], probably resulting in the use of short-chain fatty acids, to help maintain membrane fluidity.

Thermal stress induced by exposure to low temperatures affects the cell osmotic balance, resulting in a large cytoplasmic water efflux similar to the one occurring under salt stress. Therefore, one of the mechanisms used by the microorganisms to protect themselves from osmotic imbalance and intracellular ice formation consists of accumulating compatible solutes inside the cell [11, 39]. Thermal differential expression of membrane proteins associated with the transport of such solutes was observed in bacteria native to cold environments compared to other bacteria [28, 42]. *E. antarcticum* has several genes that encode membrane proteins associated with the transport of compatible solutes; however, only one of them was overexpressed in the cold condition (Table 2).

We also found expressed genes whose products participate in the carotenoid synthesis pathway. The presence of such transcripts indicates that the orange color of *E. antarcticum* B7 colonies (data not shown) is due to the production of such pigments, which has been frequently reported in polar microorganisms [12, 19]. In these microorganisms, the pigments might contribute to the adjustment of membrane fluidity to low temperatures [49] as well as to protection against ultraviolet (UV) radiation. Therefore, they may prevent cell oxidation [50]. Such a protective mechanism is most likely highly relevant for *E. antarcticum* B7 survival in Antarctica, which exhibited one of the highest temperature rise rates on Earth in the last 50 years [51].

#### Other remarks

The energy metabolism of *E. antarcticum* exhibits changes similar to the ones shown for *E. sibiricum* [40], while the high solubility of O_2_ at low temperatures is one of the reasons that led us to believe that the metabolism of this bacterial species is mainly aerobic. Although pyruvate molecules are transformed into large amounts of acetyl-CoA through the action of the pyruvate-dehydrogenase complex (Table  2), the final pathway of aerobic energy production involving the cytochrome c oxidase is repressed. This effect might be due to the low metabolic rates exhibited by cells in cold environments [40]. As the genes associated with anaerobic fermentation are also repressed, the excess acetyl-CoA molecules are probably used in the synthesis of amino acids or fatty acid molecules, the latter being extremely relevant to thermal adaptation as cited above in cell membrane adaptation topic.

Enzymes involved in the combat against oxidative stress, such as peroxiredoxin and superoxide dismutase, were repressed at 0°C compared to 37°C. The latter was differentially expressed in the proteome, while its log2FC value in the transcriptome was not significant. Such repression may occur due to the low concentrations of superoxide radicals and reactive oxygen species that are produced during aerobic metabolism, which is repressed at 0°C compared to warmer temperatures. Additionally, several genes organized in operons and related to purines and pyrimidines synthesis were repressed at 0°C compared to higher temperatures. Frank et al., 2011 [34] found that *P. putida* shuts down the transcription of such operons following exposure to cold. A total of 84 genes encoding hypothetical proteins were differentially expressed according to both platforms (Additional file 3: Table S1), which points to the need to improve the understanding of the adaptive mechanisms exhibited by psychrotrophic organisms.

## Conclusions

Temperature is a critical parameter to determine life distribution on earth extreme environments, and its habitat compatibility is finally determined by the intrinsic genetic architecture [3]. The combined approaches used in the present study allowed us to verify the molecular mechanisms of cold adaptation primarily manifested as changes in gene expression, and secondarily as differential protein expression. Two next-generation technologies, with different sequencing depth, yielded a satisfactory coverage and similar transcriptomics profiles, which was also endorsed by the proteomics results. Our findings suggest that *E. antarcticum* B7 exhibits a complex regulatory network governing gene expression, that ensure its survival and growth in environments exhibiting wide temperature variations, as a psychrotrophic organism. A large number of genes were identified as differentially expressed among the two growth temperature conditions, 0°C and 37°C, it were probably related to maintain the integrity of macromolecules and the biological process, preserving the cell structure and function. To adapt to low temperatures, this bacteria overexpresses four of the six cold-shock proteins genes present in its genome, which were the most abundant transcripts, as well as the proteins identified at 0°C. These findings validate the use of multireads and even permitted to evidence post-translational protein modifications. Together with the increase of these homologues, a parallel regulation of other proteins might also contribute for the efficient transcription, translation and protein folding process. Moreover, the broad molecular cold adaptation process require modifications at membrane level, with changes in lipid composition and/or in the capture of compatible solutes; at energy production, reducing the aerobic metabolism; and probably at cell communication under stress conditions, through chemotaxis and motility. The variation in expression of genes encoding hypothetical proteins, and alternative sigma factors without regulons descripted in *E. antarcticum* B7, highlight the necessity for additional investigation of this organism. Further integrated analyses are required to understand the switch control of the thermo sensitive pathways, helping to unveil the lifestyle of the bacterial species isolated from the Antarctica region.

### Availability of supporting data

The data sets supporting the results of this article are available in the Sequence Read Archive (SRA) repository under the accession number SRP045761.

## Electronic supplementary material

Additional file 1: **Electronic file (.ppt) containing two additional figures.** **Figure S1** – Definition of the positions of reads with more than one genome mapping possibility (multireads)**.** (A) Mapping criterion used when the alignment was performed including a parameter to discard multireads. (A.1) Different genes with one conserved region; reads aligned to this region are discarded. (A.2) Genes present in more than one copy; all the reads aligned to these genes are discarded. (B) Mapping criterion used when the alignment was performed including a parameter to include multireads (N possible mappings ≤10). (B.1) Different genes with one conserved region; reads aligned to this region are distributed between both genes as a function of the expression of their non-conserved regions. (B.2) Genes present in more than one copy; reads aligned to these genes are equally distributed between both copies. **Figure S2** – Contribution of multireads to RPKM final value. The graphic depicts the percent contribution of multireads to the final RPKM values of the transcripts expressed in the libraries sequenced on the SOLiD platform at 0°C and 37°C (R1-0 and R1-37, respectively). (PPTX 98 KB)

Additional file 2: **Electronic file (.tiff) with an image of the initial two-dimensional gel stained with colloidal Coomassie.** Two-dimensional gel obtained with a 24 cm pH 3–11 NL strip digitized using Image Scanner II (GE Healthcare, Sweden). Most of the spots formed in the two-dimensional gel were detected in the acidic range. (TIFF 878 KB)

Additional file 3: **Excel file containing two additional tables:** **Table S1- RNA-Seq analysis of** ***Exiguobacterium antarcticum*** **B7 growth at 0°C and 37°C.** Summary of the RNA-Seq data corresponding to all the replicates sequenced using SOLiD or Ion Torrent. **Table S2** – Proteins identified by MS/MS. Each identified spot corresponds to a match ID different from the gel. The Mascot score, gene, Locus Tag, accession number, protein name, relative volume, fold change, theoretical pI, MW and identified peptides of each protein are described in the table. (XLSX 734 KB)

Additional file 4: **Contains two additional tables: Table S3 – Expression of genes encoding** ***E. antarcticum cold shock proteins*** **(Csp).** Comparison of the results of Csp-encoding gene expression with or without multireads. **Table S4** – Expression of the genes included in the operon for flagellar synthesis and bacterial chemotaxis. The 31 operon genes were expressed more highly at 0°C than at 37°C; this operon includes the gene encoding the sigma D alternative factor (highlighted). (PDF 41 KB)
