# Supplementary figures and images for: Omics profiles used to evaluate the gene expression of Exiguobacterium antarcticum B7 during cold adaptation
Source: BMC Genomics. 2014 Nov 18;15(1):986. doi: 10.1186/1471-2164-15-986 (PMC4247613; doi:10.1186/1471-2164-15-986)

## Slide 1
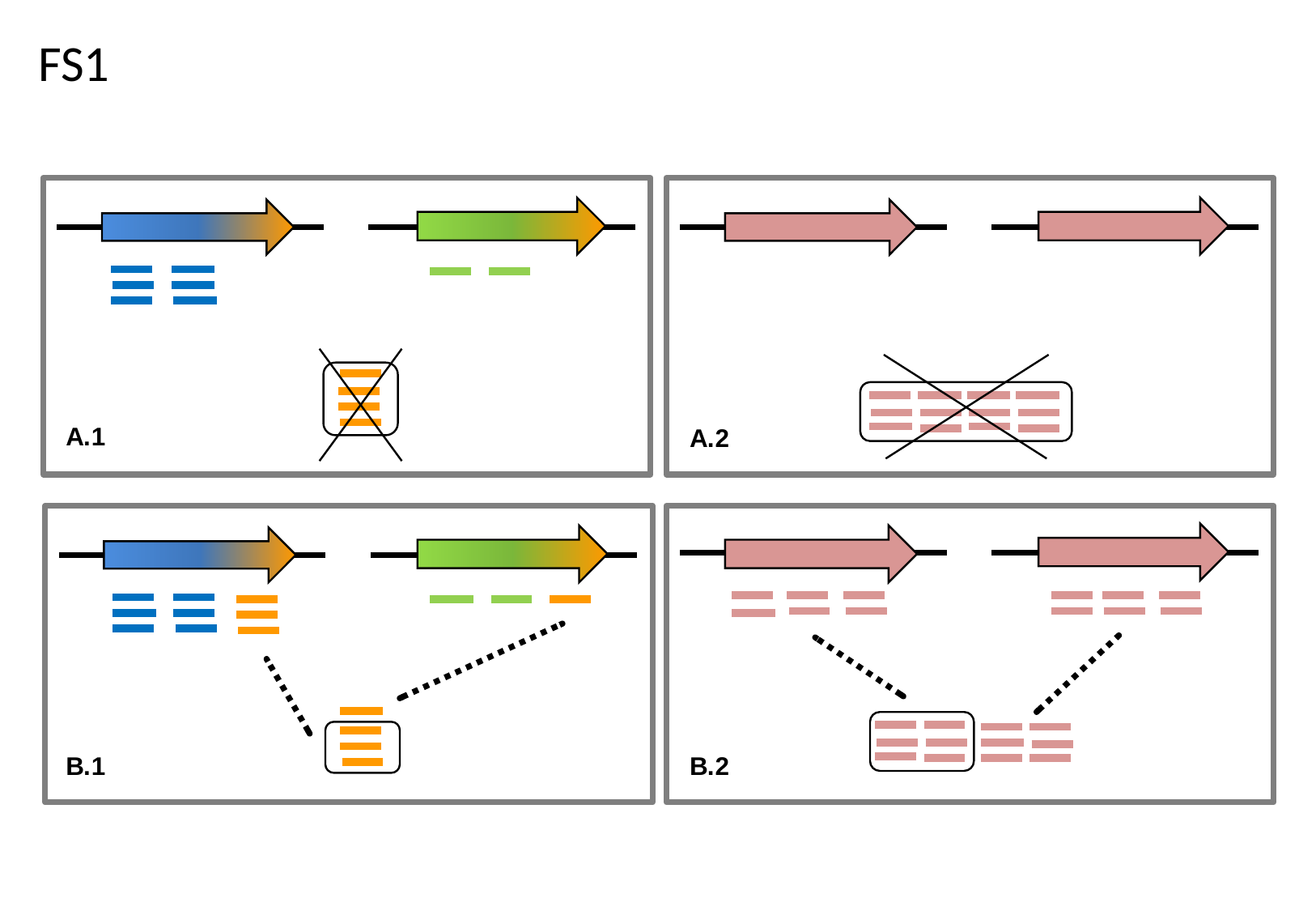

FS1

## Slide 2
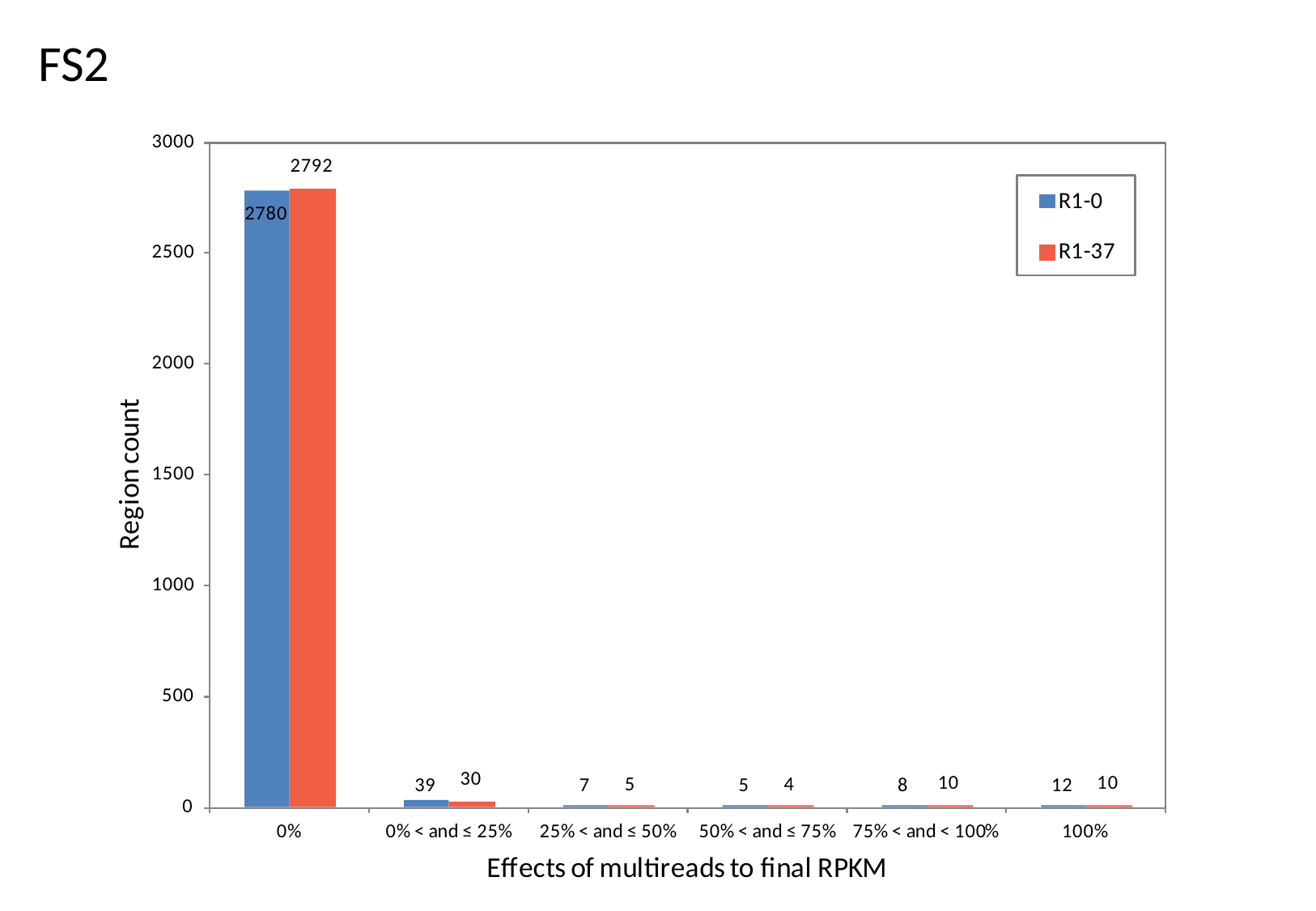

FS2

Supplement: Supplementary file 1 — Additional file 1: Electronic file (.ppt) containing two additional figures. Figure S1 – Definition of the positions of reads with more than one genome mapping possibility (multireads). (A) Mapping criterion used when the alignment was performed including a parameter to discard multireads. (A.1) Different genes with one conserved region; reads aligned to this region are discarded. (A.2) Genes present in more than one copy; all the reads aligned to these genes are discarded. (B) Mapping criterion used when the alignment was performed including a parameter to include multireads (N possible mappings ≤10). (B.1) Different genes with one conserved region; reads aligned to this region are distributed between both genes as a function of the expression of their non-conserved regions. (B.2) Genes present in more than one copy; reads aligned to these genes are equally distributed between both copies. Figure S2 – Contribution of multireads to RPKM final value. The graphic depicts the percent contribution of multireads to the final RPKM values of the transcripts expressed in the libraries sequenced on the SOLiD platform at 0°C and 37°C (R1-0 and R1-37, respectively). (PPTX 98 KB) [file 12864_2014_6691_MOESM1_ESM.pptx]

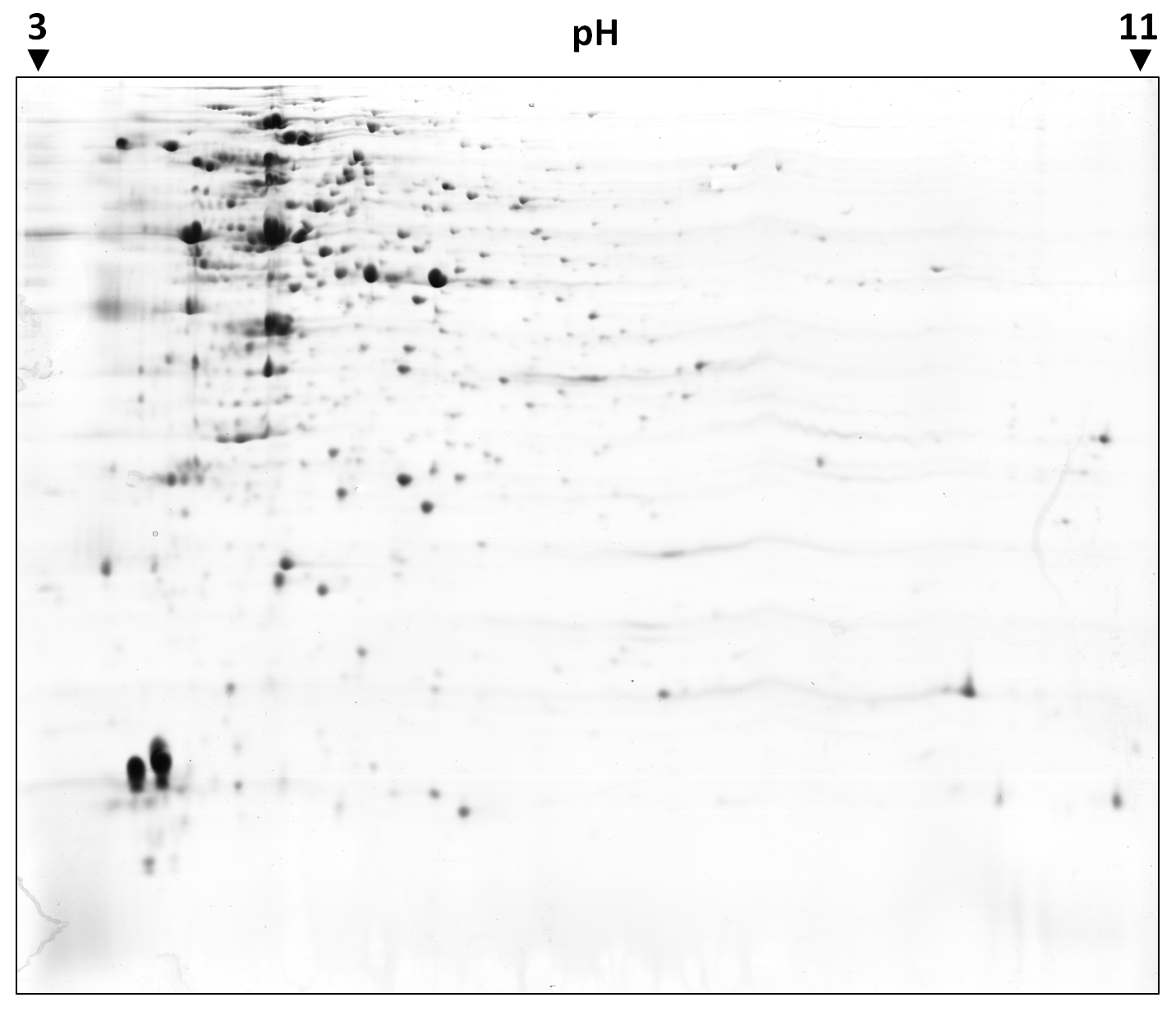

Supplement: Supplementary file 2 — Additional file 2: Electronic file (.tiff) with an image of the initial two-dimensional gel stained with colloidal Coomassie. Two-dimensional gel obtained with a 24 cm pH 3–11 NL strip digitized using Image Scanner II (GE Healthcare, Sweden). Most of the spots formed in the two-dimensional gel were detected in the acidic range. (TIFF 878 KB) [file 12864_2014_6691_MOESM2_ESM.tiff]
