# Supplementary material for: Omics profiles used to evaluate the gene expression of Exiguobacterium antarcticum B7 during cold adaptation
Source: BMC Genomics. 2014 Nov 18;15(1):986. doi: 10.1186/1471-2164-15-986 (PMC4247613; doi:10.1186/1471-2164-15-986)
Supplement: Supplementary file 4 — Additional file 4: Contains two additional tables: Table S3 – Expression of genes encoding E. antarcticum cold shock proteins (Csp). Comparison of the results of Csp-encoding gene expression with or without multireads. Table S4 – Expression of the genes included in the operon for flagellar synthesis and bacterial chemotaxis. The 31 operon genes were expressed more highly at 0°C than at 37°C; this operon includes the gene encoding the sigma D alternative factor (highlighted). (PDF 41 KB) [file 12864_2014_6691_MOESM4_ESM.pdf]

Table S3

Gene ID	Product	DNA Strand	SOLiD* (R1-0 vs R1-37) R1-0 reads	R1-37 reads	R1-0 RPKM	R1-37 RPKM	log2 FC	Ion Torrent (R2,3-0 vs R2,3-37) log2 FC
Eab7_1546	Cold shock protein Csp1	+	40181	2678	18189.97	2962.30	2.61	1.94
			[0]	[0]	[0,00]	[0,00]		
Eab7_1547	Cold shock protein Csp2	+	103503	3471	46855.90	3839.49	3.6	2.16
			[38]	[3]	[17,56]	[3,33]		
Eab7_1548	Cold shock protein Csp3	+	40442	2634	18308.13	2913.63	2.65	2.30
			[0]	[0]	[0,00]	[0,00]		
Eab7_1549	Cold shock protein Csp4	+	30512	2073	13812.81	2293.07	2.59	2.46
			[0]	[0]	[0,00]	[0,00]		
Eab7_2272	Cold shock protein Csp5	-	893	2591	404.26	2866.06	-2.8	-1.06
			[869]	[2591]	[401,50]	[2876,37]		
Eab7_2747	Cold-shock DNA-binding domain protein	-	5502	8102	2490.76	8962.12	-1.84	-1.28
			[5490]	[8100]	[2536,55]	[8992,14]		

* The values of total reads and RPKM obtained from the SOLiD system, counting (above) or discarding (lower brackets) the multireads.

Table S4

Gene ID	Product	SOLiD (R1-0 vs R1-37)	Ion Torrent (R2,3-0 vs R2,3-37)
		log_2FC	log_2FC
Eab7_1703	Hypothetical protein	1.93	3.15
sigD	**RNA polymerase sigma-D factor**	2.6	2.14
cheD	Chemoreceptor glutamine deamidase CheD	2.88	3
cheC	CheY-P phosphatase CheC	2.7	2.57
cheW	Chemotaxis protein CheW	2.5	2.7
cheA	Chemotaxis protein CheA	2.7	3.2
ylxH	ylxH protein	3	3
flhF	Flagellar biosynthesis protein flhF	2.46	3.8
flhA	Flagellar biosynthesis protein flhA	2.5	2.72
flhB	Flagellar biosynthetic protein flhB	2	3.32
fliR	Flagellar biosynthetic protein fliR	2.93	2
fliQ	Flagellar biosynthetic protein FliQ	3.33	10.5
fliP	Flagellar biosynthetic protein fliP	2.67	2.32
fliZ	Flagellar biosynthetic protein fliZ	2	2.27
cheY	Chemotaxis protein CheY	2.52	5
fliY	Flagellar motor switch phosphatase FliY	2.29	3.12
fliM	Flagellar motor switch protein FliM	2.25	2.11
fliL	Flagellar basal body-associated protein FliL	2	2.94
flbD	Flagellar protein flbD	2.33	2.67
flgG	Flagellar basal-body rod protein flgG	2.18	2.99
flgD	Flagellar hook capping protein	2.2	2.97
fliK	Flagellar hook-length control protein	2.69	2.96
Eab7_1725	MgtE intracellular region	2.3	2.19
fliJ	Flagellar biosynthesis chaperone FliJ	2.5	2.42
fliI	Flagellum-specific ATP synthase	2	2.5
fliH	Flagellar assembly protein fliH	2.1	2.52
fliG	Flagellar motor switch protein FliG	1.4	2.35
fliF	Flagellar M-ring protein	1.36	2
fliE	Flagellar hook-basal body complex protein FliE	0.98	<u>0.74</u>
flgC	Flagellar basal-body rod protein flgC	1.63	1.77
flgB	Flagellar basal-body rod protein flgB	1.16	1.54

Log2FC underlined correspond to non-significant P-value (P-value>0.001).
